# Timescale of tumor volume of a young breast cancer patient with luminal B subtype: A case report

**DOI:** 10.1097/MD.0000000000017659

**Published:** 2019-10-25

**Authors:** Xiaoyun Mao, Ming Zhou, Chuifeng Fan, Bo Chen, Feng Jin

**Affiliations:** a Department of Breast Surgery, The First Affiliated Hospital of China Medical University, Shenyang, Liaoning, People's Republic of China; b Institute of Mathematics, University of Rostock, Ulmenstrasse 69, Haus 3, Rostock, Germany; c Department of Pathology, The First Affiliated Hospital and College of Basic Medical Sciences of China Medical University, Shenyang, Liaoning, People's Republic of China.

**Keywords:** breast cancer, timescale, tumor volume

## Abstract

**Rationale:**

It is largely unknown about the tumor growth of breast cancer naturally. We devised and analyzed an appropriate mathematical tool of the equations that describe how fast tumors grow without treatment on the basis of the ellipsoid shape of solid breast cancer.

**Patient concerns:**

A 31-year-old woman presented with a painless palpable lump in her left breast for 5 months.

**Diagnosis:**

Infiltrated ductal breast cancer (histologic grade II) of luminal B

**Interventions:**

The patient did not receive any therapy due to her private reasons for 2 years, the analysis of the tumor volume growth was done regarding the growth rate of the tumor in the absence of intervention.

**Outcomes:**

After 2 years of diagnosis of breast cancer, the tumor mass occupied the whole left breast with skin implanted and nipple abnormality. As this case indicated that the tumor's early growth rate was very slow. When the tumor volume reached 300 cm^3^, its fast growth began without treatment. Later growth approached the maximum, when the tumor volume was more than 800 cm^3^.

**Lessons:**

The tumor growth is segmental without therapy. Early diagnosis and treatment is the key to good prognosis for every breast cancer patient.

## 1 Introduction

How about tumor growth in breast cancer? How long does a tumor solid get its tumor volume in breast cancer? Researchers have always assumed tumor growth from the medical history described by the patient. How about the advanced timescale of the tumor volume of a breast cancer patient without treatment? It is largely unknown, but it is very important. The mathematics of tumor growth can lead to the discovery of just changing the curve of tumor growth by chemotherapy or radiotherapy accurately. Traditionally, the longest diameter measurement is regarded to represent the risk of cancer metastasis and the probability of distant recurrences, an integral component widely used in the TNM staging system.^[1]^ Maybe it is more accurate to estimate the tumor burden or tumor growth by calculating its volume instead of the traditional longest diameter. Tumor is a 3D object that can be measured along three axes. The three-dimensional fundamental shape of tumors is assumed to be ellipsoid in different studies. In a previous study, tumor volume is approximated using a circumscribing ellipsoid.^[2–4]^ We devised and analyzed an appropriate mathematical tool of the equations that describe how fast tumors grow without treatment. We describe the timescale of tumor volume of a young case of primary luminal B breast cancer without treatment.

## 2 Case report

**Figure 1:**
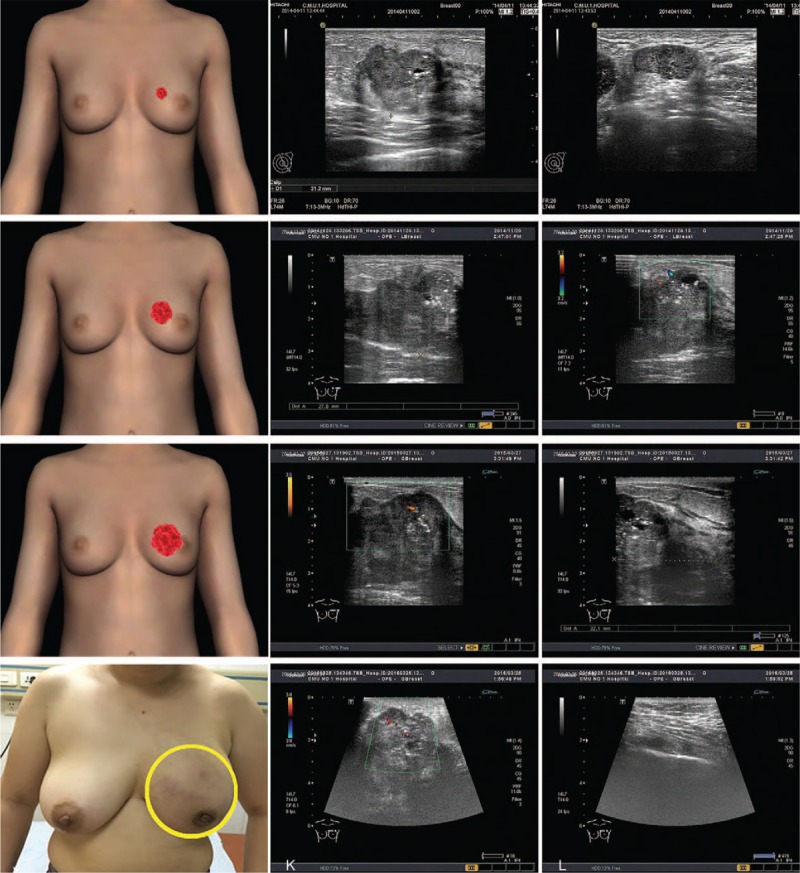
Tumor solid in this patient. (A) Tumor mass model on April 11, 2014. The tumor mass was 2.93 × 2.79 × 2.12 cm at 11 o’clock position, 2.6 cm from the nipple. (B and C) Tumor mass on ultrasound in April 11, 2014. (D) Tumor mass on November 20, 2014. It was 4.56 × 2.09 × 2.78 cm as detected by ultrasound. (E and F) Tumor mass on ultrasound on November 20, 2014. (G) Tumor mass model on March 27, 2015. It was 7.3 × 6.0 × 4.0 cm as detected by ultrasound. (H and I) Tumor mass on ultrasound on March 27, 2015. (J) Tumor mass in this patient on March 28, 2016. The size of the tumor was 16 × 13.5 × 12 cm, which occupied the whole left breast with skin implanted and nipple abnormality. (K and L) Tumor mass on ultrasound on March 25, 2016.

**Figure 2:**
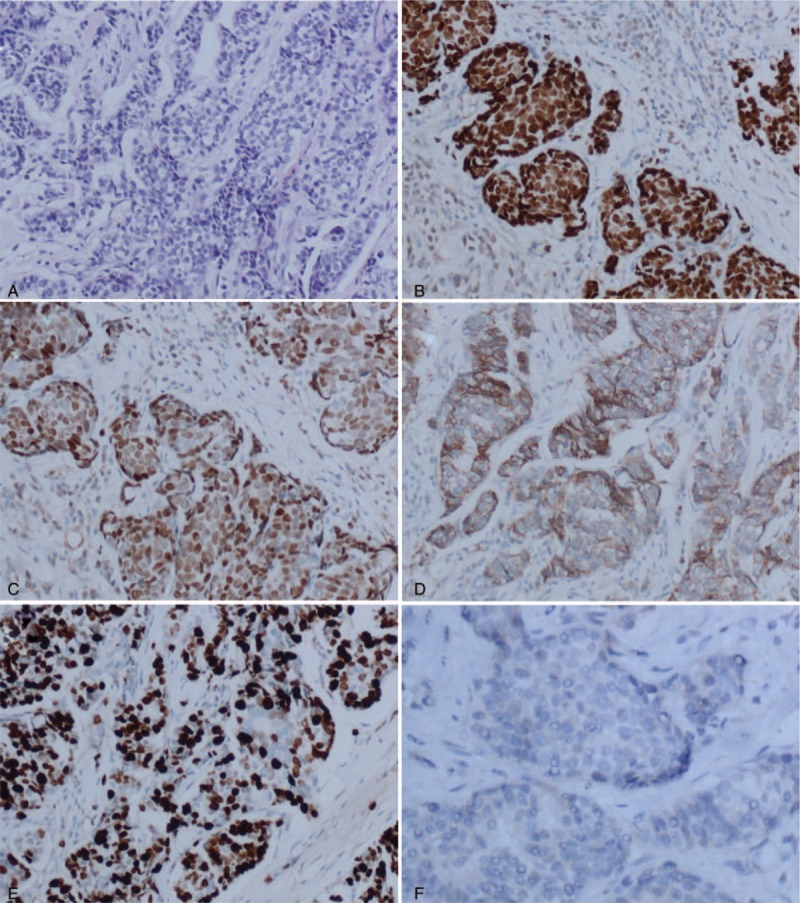
Pathological picture of the tumor in this patient. (A) Hematoxylin-eosin staining showed the infiltrated ductal breast cancer of the core biopsy in this case, intermediate nuclear grade (histologic grade II). (B) Immunohistochemical staining of ER in the core biopsy. ER positive rate was 80%. (C) Immunohistochemical staining of PR. PR positive rate was 80%. (D) Immunohistochemical staining showed HER-2 2+. (E) Ki67 positive rate was 50%. (F) Expression of p63 was negative. Original magnification: all × 200.

The patient was a 31-year-old yellow race, nonporous, female who presented with complaints of a painless palpable lump in the upper inner quadrant of her left breast for 5 months in April 2014. The size of the mass enlarged gradually from 2 × 2 × 2 to 3.0 × 3.0 × 2.0 cm over the 3 months at that time. Ultrasound demonstrated a solid mass of 2.93 × 2.79 × 2.12 cm at 11‘o’clock position, 2.6 cm from the nipple. The solid mass was categorized as BRIRADS 5 according to the standards of the BIRADS-US. The ultrasound of the axilla revealed some enlarged left axilla lymph node, in which the biggest one was 2.81 × 1.08 cm and categorized as BIRADS-US 5 (Fig. 1). A core biopsy was taken from the solid mass of her left breast and reported as infiltrated ductal breast cancer (histologic grade II) by pathologists (Fig. 2). Immunohistochemically, the results were reported to be ER positive 80%, PR positive 85%, HER-2 2+ (HER-2 negative amplification by FISH), and Ki67 positive 30% (Fig. 2). It was considered as luminal B of the breast cancer subtype according to the St. Gallen International Expert Consensus.^[5]^ However, she did not receive any therapy due to her private reasons. Seven months later, the solid mass enlarged from 2.93 × 2.79 × 2.12 to 4.56 × 2.09 × 2.78 cm, as detected by ultrasound on November 20, 2014 (Fig. 1). On March 27, 2015, it enlarged to 7.3 × 6.0 × 4.0 cm as detected by ultrasound. On March 25, 2016, the size of the tumor was already 16 × 13.5 × 12 cm, which occupied the whole left breast with skin implanted and nipple abnormality (Fig. 1).

## 3 Discussion

**Figure 3:**
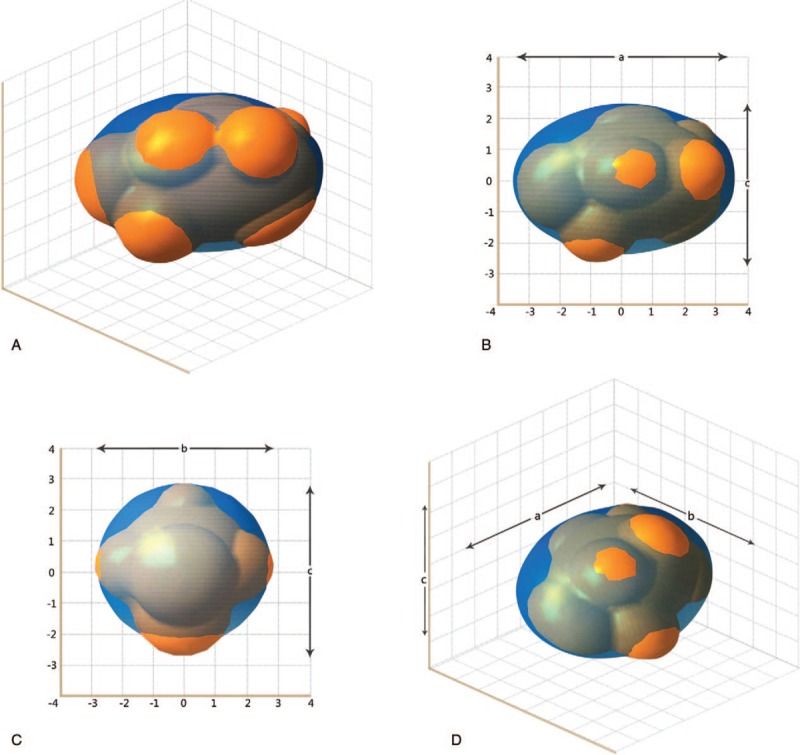
Ellipsoid model of the tumor solid. We estimated the volume of the tumor solid with ellipsoidal estimation. (A) Three dimensions of the tumor. (B) a- and c-diameters of the tumor. (C) b- and c-diameters of the tumor. (D) Irregular-shaped tumor with ellipsoidal estimation.

**Figure 4:**
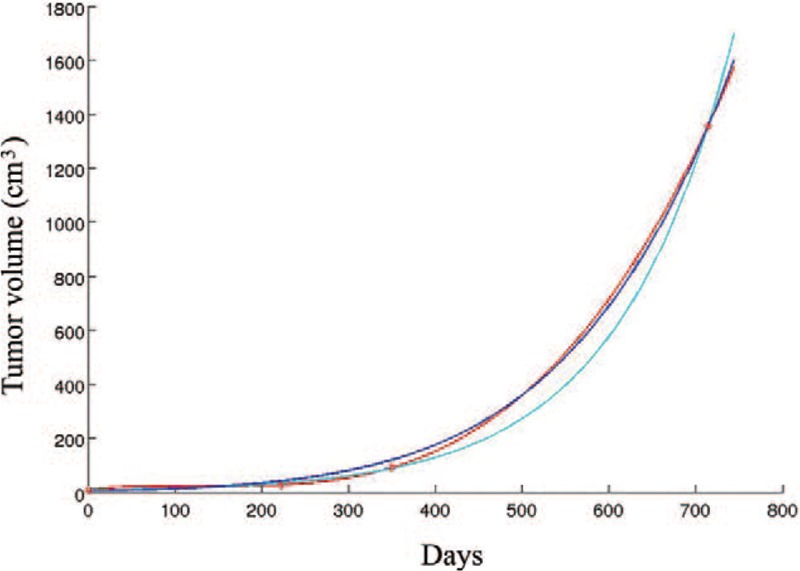
Curves for tumor volume data. Red curve: cubic interpolating polynomial c_0_ + c_1_∗t + c_2_∗t^2^ + c_3_∗t^3^ with c_0_ = 9.074000, c_1_ = 3.359018e−01, c_2_ = −2.649229e-03, and c_3_ = 6.755122e-06. Cyan curve: simple exponential function c∗exp(a∗t), where c = 6.407120 and a = 7.501095e−03. Blue curve: Gompertz function n∗exp(ln(N/n)∗(1−exp(−a∗t))), where a = 9.047358e−04, n = 4.810605, and N = 6.768107e + 05.

**Figure 5:**
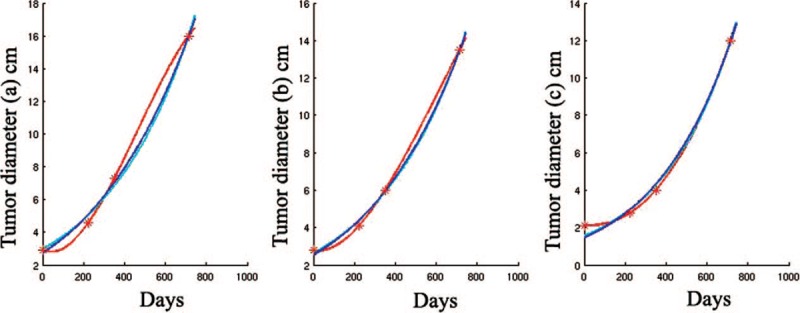
Curves for tumor diameter data (axis lengths). The red curve represents the result of the polynomial interpolation. The cyan and blue curves represent the results from solving nonlinear least-squares problems with respect to exponential functions and Gompertz functions, respectively.

**Figure 6:**
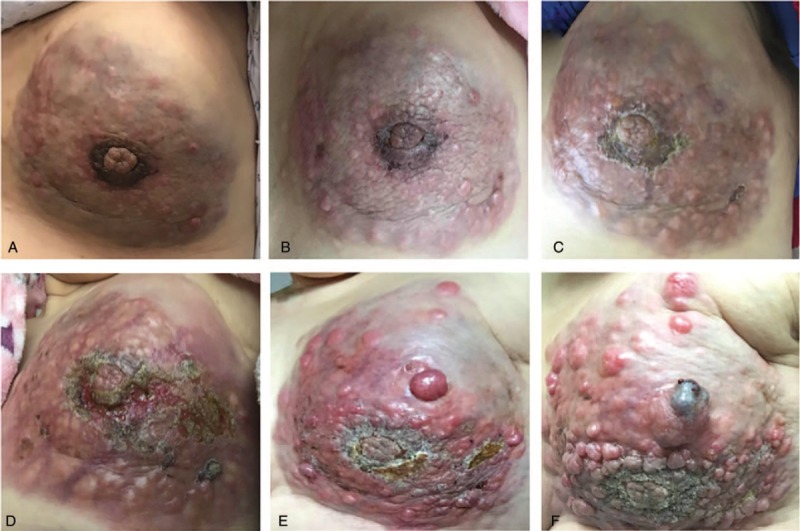
Tumor solid in this patient with treatment. (A) Tumor mass on Oct 27, 2017. (B) Tumor mass on Nov 20, 2017. (C) Tumor mass on Dec 22, 2017. (D) Tumor mass on Feb 2, 2018. (E) Tumor mass on Mar 18, 2018. (F) Tumor mass on May 18, 2018.

The three-dimensional fundamental shape of solid tumors is assumed to be a hemi-ellipsoid in different solid tumors,^[6]^ such as breast cancer,^[2,7–9]^ hepatocellular cancer,^[10]^ prostate cancer^[11]^ and so on. So the approximate value of the tumor volume is calculated using the formula: V = 4/3π (a/2 × b/2 × c/2), where a, b, and c are the measured axis lengths from the data shown in Fig. 3. We devised and analyzed equations that describe how fast tumors grow without treatment. In our research, the interpolation gives a cubic polynomial c_0_ + c_1_∗t + c_2_∗t^2^ + c_3_∗t^3^ with the coefficients c_0_ = 9.074000, c_1_ = 3.359018e-01, c_2_ = −2.649229e-03, and c_3_ = 6.755122e−06. The exponential function reads c∗exp(a∗t), where c = 6.407120 and a = 7.501095e−03, and the Gompertz function has the form n∗exp(ln(N/n)∗(1-exp(-a∗t))), where a = 9.047358e−04, n = 4.810605, and N = 6.768107e + 05. The tumor growth without any treatment is approximately shown in Figs. 4 and 5 with various curves. The graphs demonstrate changes in tumor volume and axis lengths over time. The curves are derived with a polynomial interpolation and by solving nonlinear least-squares problems with respect to an exponential function and a Gompertz function, respectively. They are curves for tumor diameter data which represent the results from solving nonlinear least-squares problems with respect to exponential functions and Gompertz functions, respectively (Fig. 5). As the corresponding growth curve represents, the early growth rate was relatively slow. When the tumor volume reached 300 cm^3^, its fast growth began. Later growth approached the maximum, when the tumor volume more than 800 cm^3^. Gomepetz model is one of the most frequently used sigmoid models and widely used in many aspects of biology. It was used to describe the growth of animals and plants, as well as the number or volume of bacteria and cancer cells.^[12–18]^ Laird also used Gompertz curves to fit data of the growth of tumors which had horizontal asymptote, they indicated that the observed the growth of tumor progress toward an upper limit of size, the upper limit computed for mouse fell within a relative narrow range and the size actually achieved before the death of the host was usually a high proportion of theoretical limit of growth.^[19]^ The basic idea is that the instantaneous proliferation rate of the cellular population, whose decreasing nature is due to the competition for the nutrients and the increase of the cellular population, is similar to the logistic growth rate.^[20,21]^ Gompertz curves have global upper limits. However, different tumor cells have different growth characteristics under different conditions, so the upper limits of an approximating Gompertz curve cannot be reached in some practical cases. And we cannot obtain the Gompertz curve if the size at which the tumor was detected, or the starting size of the tumor, was incredibly small. Despite the drawbacks the merits of the Gompertz model still holds. As shown in our example, the approximation with the Gompertz function gives similar growth rates to those from the other two approximations within the practical time interval. Our result is a supplementary to the work of Laird on tumor growth. A early study had focused on the tumor growth rate and its prognosis of breast cancer by mass screen.^[22]^ Their results indicated that breast cancer doubling time was an log-normal distribution approximately and the geometric mean of doubling times was 174 days. The solid-tubular histologic type of carcinoma had the shortest geometric mean of doubling time (126 days). But the limitation of the research is some data was based on the assumption of a tumor of a detectable threshold size at previous time if they did not habe vagus or invisible shadows. This young patient was unwilling to receive any treatment when she was diagnosed with breast cancer due to private reasons. Two years later, the tumor occupied the whole left breast with skin implanted and nipple abnormality with metastasis. Our study carried several important limitations. We did not collect more clinical data of this case because she refused any treatment and missed the best time for therapy. Later, the patient received chemotherapy from April 2016, but the local tumor in breast progressed (Fig. 6), the bone and visceral metastasis progressed also. She died of the multiple metastasis of breast cancer at May, 2019.

In conclusion, breast cancer tumor is segmental without valid therapy. As this case indicated that the tumor's early growth rate was very slow. When the tumor volume reached 300 cm^3^, the fast growth began. Later growth approached the maximum, when the tumor volume was more than 800 cm^3^. Early diagnosis and treatment is the key to good prognosis for every breast cancer patient.

## Author contributions

**Conceptualization:** Xiaoyun Mao.

**Data curation:** Chuifeng Fan.

**Formal analysis:** Ming Zhou, Feng Jin.

**Investigation:** Xiaoyun Mao, Feng Jin.

**Methodology:** Ming Zhou, Bo Chen, Feng Jin.

**Project administration:** Chuifeng Fan, Bo Chen, Feng Jin.

**Resources:** Chuifeng Fan, Bo Chen.

**Supervision:** Feng Jin.

**Writing – original draft:** Xiaoyun Mao.

**Writing – review & editing:** Bo Chen, Feng Jin.
